# The Imperforate Anus Psychosocial Questionnaire (IAPSQ): Its construction and psychometric properties

**DOI:** 10.1186/1753-2000-3-15

**Published:** 2009-05-14

**Authors:** Margret Nisell, Ulf Brodin, Kyllike Christensson, Per-Anders Rydelius

**Affiliations:** 1 Child and Adolescent Psychiatric Unit, Department of Woman and Child Health, Karolinska Institutet, Stockholm, Sweden; 2 The Medical Statistics Unit, Department for Learning, Informatics, Management and Ethics (LIME), Karolinska Institutet, Stockholm, Sweden; 3 Reproductive and Perinatal Health Care Unit, Department of Woman and Child Health, Karolinska Institutet, Stockholm, Sweden

## Abstract

The origin of the present study was to develop the liaison work between the disciplines of child and adolescent psychiatry and paediatric surgery and nursing, so as to improve the quality of treatment and care of a group of children with imperforate anus (IA) and their families. Imperforate anus is a congenital disease involving a deformity of the anorectum. The early surgery and invasive follow-up treatment associated with IA may affect the child psychosocially, including the child-parent relationship. By developing and testing a questionnaire for children born with anorectal anomalies, a tool for measuring psychosocial functioning can be realized.

First, a literature review on "Imperforate Anus" was performed. Second, an exploratory interview study was conducted with patients/adolescents with IA and their parents. The findings from these interviews were the foundation for construction of the questionnaire. The Imperforate Anus Psychosocial Questionnaire (IAPSQ) was tested and revised three times before its completion. It contains 45 items on Likert scales. A total of 87 children completed the IAPSQ: 25 children with IA and two comparison groups. Face and content validity were considered. The Rasch approach, an item response theory model, was used to evaluate the psychometric properties of the IAPSQ, where item difficulty and person ability are concurrently approximated.

The findings of the Rasch analysis revealed that the psychological dimension was reasonable, and that person reliability (0.83) was moderate and item reliability (0.95) was sufficient. The social dimension showed satisfactory item reliability (0.87). The person reliability (0.52) of the social dimension was weak. Content validity seemed to be established and construct validity was recognized on the psychological dimension.

The IAPSQ provides a reasonably valid and reliable measure of psychosocial functioning for clinical use among children with IA, although some revisions are suggested for the next version of the IAPSQ. By using the Rasch model, we discovered that specific items should be discarded and other items should be reformulated to make the questionnaire more "on target". The social dimension has to be expanded with further items to reasonably capture a social dimension.

## Background

The psychosocial functioning of children born with physical and mental handicaps depends not only on the handicap, but also on environmental factors and support, as most children's health and well-being are closely linked to their parents' physical, emotional and social health and to their social environment [1]. According to Geist and Grdisa [2], psychosocial issues are vital to adaptation and to the overall outcome for children with chronic conditions and their families. When healthy children were asked about the most important aspects of quality of life, younger children prioritized family functioning, while older children chose their own social functioning as the most important aspect [3]. Psychosocial functioning and quality of life can be regarded as interchangeable terms, and if not identical, they seem to be closely related. When measuring quality of life issues, physical, psychological (including emotional and cognitive) and social health dimensions are defined [4]. Psychosocial functioning was demonstrated to be the most important factor explaining quality of life in patients with anorectal malformations [5].

In paediatric research on younger children, assessments made by parents, i.e. proxy ratings, are commonly used. Parents are viewed as suitable sources of information on their child's illness and quality of life [6-8]. However, there is a call for valid and reliable child self-report instruments [9]. As the child is the person of interest, it is essential to obtain his/her self-ratings, and although proxy versions are useful, the child should be considered the primary informant regarding his/her own health and related quality of life [10].

Our interest was in children with Imperforate Anus (IA). Imperforate Anus is a congenital disease involving a deformity of the anorectum. This congenital anomaly and its consequences require early surgery and invasive follow-up treatment that may affect the child psychosocially, including the child-parent relationship and interaction. A reconstruction of the anus and the anorectal canal is performed when the child is 2–3 months; this is followed by anorectal dilatations to avoid strictures [11]. Functional problems, i.e. fecal incontinence and/or constipation, are common among children with IA [12,13].

Further research is needed on children with IA, as their disease-specific problems and psychosocial adjustment have not yet been fully explored. Hassink [14] described psychosocial difficulties in this group, and Meijer [15] found that children with IA often had difficulties in relationships with peers. According to Ludman, Spiz and Kiely [16], there is a link between the physical and emotional difficulties of children with IA, as many of their physical problems (i.e. odours caused by flatus and fecal incontinence) are socially unacceptable. In a study on the long-term outcome of anorectal malformations, the majority of patients/children had behavioural problems and 15% expressed suicidal thoughts, though more than half scored above average on a global hopefulness scale [17].

Liaisons between child psychiatry and paediatrics and paediatric surgery have a long history in Sweden, dating back to the 1940s [18]. Underlying our interest in developing a questionnaire for children born with anorectal anomalies that focuses on their wellbeing is our ambition to expand this liaison work into an integrated and mutual activity, in this case between the disciplines of child and adolescent psychiatry, paediatric surgery and nursing, the goal being to improve the quality of treatment and care of this group of patients and their families.

The aim of the present study was to evaluate the psychometric properties of a self-report questionnaire constructed to assess the psychological and social functioning of children with IA. This questionnaire is designed to systematically cover the wide range of features associated with the phenomenon of imperforate anus. The present study describes the construction of the questionnaire and the analytic process used to test its psychometric properties. The questionnaire is called the Imperforate Anus Psychosocial Questionnaire (IAPSQ).

## Methods

The present project was planned to evaluate the physical and mental situation of children born with IA in order to develop a multidisciplinary clinical programme to support these children and their families from birth to adult life. However, as there was no disease-specific instrument (in Swedish) for children with IA, and as there was no available generic QoL instrument (in Swedish) for children with chronic conditions, the first step was to construct a comprehensive, sound instrument for these purposes.

A literature review on "Imperforate Anus" was carried out. No questionnaire addressing the specific questions of IA was found in the literature. Thus, an exploratory interview study was designed to create *a baseline for further research *[19]. A panel consisting of two paediatric surgeons with long experience of children born with IA, a child and adolescent psychiatrist with long experience of liaising with paediatric surgery and general paediatrics, and two nurses (one with long experience of care and support given to families with children born with IA, and one from child psychiatry with long experiencing of liaising with paediatric wards) discussed the issues of concern. A number of items covering the child's well-being and adjustment, relationships to friends and school issues, physical problems related to the malformation and experiences of care were summarized and written as interview questions.

The interview questions were structured, semi-structured, and open: 88 questions for the parents and 34 questions for the children/patients. These exploratory interviews included 3 selected patients with high imperforate anus, for whom the malformation caused severe problems, and their parents. The interviews lasted from 1 to 2 hours. The individual interviews were performed by the two members of the panel with experience of child and adolescent psychiatry; they were considered neutral interviewers and had had no previous contact with any of the interviewees.

Immediately after the interviews, the interviewees were asked to add any questions of concern that may have been missed during the interviews. Further, the interviewees were given the interview questions and were asked to read them later on. Two weeks later, they were contacted by telephone and asked whether there were any inappropriate questions that should be omitted or whether any questions were missed that should be added. None of the interviewees wanted to exclude, change or add any questions or concerns.

The interviews were analysed, and the interview findings served as the foundation for identifying variables of interest that were to be operationalized in questions formulated for the study-specific questionnaire. The aim was to design a questionnaire for children born with anorectal anomalies and their parents that could evaluate their psychosocial functioning, their adjustment to the disorder and their experiences of the care provided by the staff at the department for paediatric surgery. Responses to such questions were expected to facilitate the improvement of treatment and care.

### Questionnaire construction procedure

The variables identified from the exploratory interviews revealed the issues of concern to children with IA and their parents, thus the issues essential to creating the questionnaire items. Yet again, the panel of specialists in paediatric surgery and child and adolescent psychiatry assembled to formulate questions, now for the questionnaire.

Operationalization of the questionnaire involved a review of generic questionnaires, such as Quality of Life questionnaires, used to measure psychosocial factors in children. Moreover, experts in special pedagogy were contacted and consulted to ensure the construction of age-appropriate scales and questions. The questionnaire was intended to examine and measure psychosocial functioning in children with IA. The questionnaire included two main aspects:

1) *Psychological *issues, such as emotional well-being including cognition and self-determination, and 2) *Social *issues, items covering relationships with friends and family as well as at school. Physical issues, such as the child's bowel function, were also included. Another topic derived from the interviews was experiences of care and treatment.

#### Testing the Questionnaire

Three subsequent tests were performed on the children's questionnaire, comprising reports from 14 children. All informants who were asked to participate in the questionnaire testing were willing to do so, and all of them returned the questionnaires. All informants were assured confidentiality.

The first test addressed two "ordinary" healthy children, 7 and 8 years of age, who were not involved in any of the three prospective groups for the main study. The intention was to ensure the soundness, comprehensiveness and age appropriateness of the questions for children. These two children easily completed the questionnaire, which indicated that we were on the right track.

The second questionnaire test consisted of 9 children from the 3 categories that were to be involved in the main study: 3 children with IA, 3 children with juvenile chronic arthritis (JCA), and 3 children with no chronic condition (NCC), along with their respective parents. The families in the index group, the IA group, were contacted through the hospital register at the outpatient clinic for paediatric surgery. Three families of a child born in 1993 who had been operated on for high or intermediate IA were chosen.

The children with JCA and their parents were contacted with the help of the staff and hospital register at the outpatient clinic for rheumatic children. Three families of a child with JCA were selected, contacted and informed about the study. The children were born in 1993, had had JCA since 2 years of age, and had received joint injections before the age of 4 years.

The next group participating in the second test consisted of 3 children without a chronic condition (NCC) and their parents. They were found through the day surgery clinic and had undergone minor surgery (e.g. for a hernia). The children were born in 1993 and were consecutively chosen.

An appraisal and systematic survey of the completed test questionnaires was carried out. The outcome of the second testing showed lack of validity for some items in the child questionnaire. The responses from the children with NCC showed difficulties in "understanding" what the word *condition *meant, here in relation to the minor surgical procedure they had undergone. Some of the items associated with the word *condition *had been ignored and left unanswered. Consequently, small clarifying linguistic revisions were made for the third questionnaire test.

The third and last test of the questionnaire involved 3 children with NCC. The children's responses to the third test revealed no problematic items, and therefore no additional revisions were necessary.

It is worth noting that all children participating in the pilot studies were born in 1993. The questionnaire was tested on somewhat younger children because the already small number of children with IA in the specific age group that was the focus of the main study should not be reduced.

#### Resulting Questionnaire

The completed questionnaire, the Imperforate Anus Psychosocial Questionnaire, the IAPSQ, consisted of 45 items covering the following domains: Emotional (12 items), Emotional/Cognition (7 items), Self-determination (5 items), Social relationships and School (13 items) and Physical function (4 items) and Experiences of care (4 items). The IAPSQ comprised data on the child's specific condition, sex and age.

In the opening section, the first 24 items were designed as a pictogram featuring five faces with various expressions. In this section, the items covering the above-mentioned domains were mixed. The five faces in the pictogram depicted the most negative answer to the left and the most positive answer to the right, always in the same order. The child was requested to mark the most reasonable answer (face). The other part of the IAPSQ, items 25–45, complemented the first part and was divided into (labelled) domains. Except for one dichotomous item/question in the second part of IAPSQ, 20 items were to be answered on a five-point Likert scale with different alternative answers/anchor words, depending on the question posed. The five possible answers on the Likert scale were positioned so that the most positive answer was to the right. In eight cases, the response alternatives were placed in the reverse order. The mixed response formats were used because some of the questions did not fit the pictogram design, and therefore Likert scales with verbal anchors were used as well. Analogous questions were posed using the two response formats to enhance the reliability. At the end of the questionnaire, the children were encouraged to comment on their experiences of answering the IAPSQ. The children were requested to fill in the IAPSQ by themselves, though if needed they could ask their parents for help.

As mentioned above, the various items in the IAPSQ were combined even though they could be classified (by researchers) into a psychological domain including three sub-domains and one social dimension. The distribution of items including the item numbers is shown in the Appendix: Table 7. The reversed scored items are marked with an asterisk. The 4 items comprising *experiences of care *and the 4 items on *physical function *were not considered in the present study.

### Participants in the Main Study

A total of 87 children completed the IAPSQ. The group of interest, the index group, contained 25 children with high and intermediate IA. These children/patients were born and had their specific surgery, the PSARP (Posterior Sagittal Anoplasty), between 1987 and 1992. The surgical technique was modified and refined at the hospital centre clinic in Stockholm, Sweden, in 1987. Children from the age of eight years were considered appropriate raters, as they were able to read and write. Originally, there were thirty children with high and intermediate IA available for the present study. Twenty-nine families with a child with IA gave their informed consent. Out of the 29 families, 25 families answered and completed the questionnaires. There were 9 boys and 16 girls involved in the study, and their mean age was 10.5 years (range 8.0–13.6). Five of the IA children had undergone additional surgery using the Malone Antegrade Continence Enema (MACE) procedure owing to severe fecal incontinence.

For reasons of comparison, the questionnaire was constructed and tested so as to be appropriate even for children without IA. Two comparison groups with experiences of clinical care were selected for participation to enhance interpretation of the findings. Comparison Group I contained children with a chronic condition: juvenile chronic arthritis (JCA). Like the IA children, this group of children had suffered from pain and emotional stress, though of another type. The inclusion criteria were an illness debut before the age of two years and joint injections before the age of 4 years. This group of children was recruited from the medical records at the outpatient clinic for paediatric rheumatism. Forty-five children and their parents were eligible. First, the families were asked by staff at the rheumatic outpatient clinic whether they were interested in receiving further information about the study. Thirty-five families agreed to be contacted for more information, and they gave their informed consent to participate. Five families did not complete the study, leaving us with 30 families involving 5 boys and 25 girls (mean age = 10.6).

Comparison Group II consisted of children who had undergone minor surgery (e.g. for a hernia), and thus who had some experience of hospital care. The families were consecutively recruited at the day surgery clinic. The children in Comparison Group II had no chronic condition (NCC) and were otherwise healthy. The families were informed about the study and asked about their interest in taking part by staff working at the day surgery clinic. If they were concerned, they were given additional information (immediately after the primary information) by the persons in charge of the study and asked about their willingness to participate. In total, fifty families of a child with NCC agreed to participate and gave their informed consent. Thirty-two families including 14 boys and 18 girls completed the study; the mean age was 10.7. All participants in the study were assured confidentiality. The study was approved by a local ethics committee.

### Assessment of Validity

In the present study, a set of psychometric properties of the IAPSQ has been considered.

There are various alternatives to assessing the validity and reliability of a questionnaire. *Face validity *concerns the extent to which the measure (the IAPSQ) reflects the content of the phenomenon (IA) and appears valid to the researcher and/or the participant. *Content validity *is concerned with the extent to which a measure sufficiently covers the full domain of a concept [20].

### Statistical methods

An Item Response Theory (IRT) approach was used to evaluate the characteristics and the usefulness of the IAPSQ in relation to its psychometric properties. The main feature of IRT is that measures are obtained from the pattern of responses rather than from total sum scores, as is typical in the classic test theory (CTT) [21,22]. A Rasch approach (an IRT model) was used to create such a measure on a one-dimensional scale. Rasch methodology is probabilistic, in that item difficulty and person ability are concurrently approximated. When the items are aggregated to form the intended dimension, IRT can identify the usefulness of an item as well as indicate whether an item contributes to forming the dimension. Unexpected answer profiles can also be identified.

The IAPSQ was constructed to reveal two main latent, non-measurable, domains. The presence of three hypothetical sub-domains of the psychological domain was also predicted. Due to the limited respondent material, we have attempted to construct a parsimonious model (i.e. one with few parameters) with a common set of category thresholds and a discrimination coefficient assumed to be equal to one. Such a simple approach also entails that the respondent's measure on the latent scale can be estimated under local independence, implying that missing values do not harm the estimation process. This assumption is further evaluated in the results section.

The Rasch model is estimated by modelling adjacent categories via a log odds expression: log (P_ink_/P_in, k-1_) = B_n _- (d_i _+ c_k_), where B_n _is the person measure and d_ι _is the item difficulty (difficulty to endorse high score) on the intended scale, and c_k _is the distance from the item difficulty to the category threshold, k = 1, 2, 3, 4.

Two Rasch models were set up: A model (a) with a common set of category thresholds, that is with (c_1_, c_2_, c_3_, c_4_) for all items, but different item difficulties. The mean item level is set equal to = 0. A model (b) with a unique set of category thresholds for each item, that is with (c_i1_, c_i2_, c_i3_, c_i4_) for item i = 1, 2,..., as well as different item difficulties. The mean item level is set equal to = 0. Model a is commonly called "the rating scale model" and model b is called "the partial credit model".

The Winsteps program 3.66 [23] was used for the model estimation process. The Winsteps program estimates the patient score on a one-dimensional interval scale and calculates the basis for evaluating the essential properties of the questionnaire items. Differences between respondent groups can also be considered. The analysis was used for the following main objectives [24].

1. Dimensionality: Do the items constituting a domain cooperate to form a predominating one-dimensional scale as intended, or do they indicate strong influence from additional latent factors?

2. Item fit: Do all items work in the same direction or are there items that do not fit into the one-dimensional trait?

3. Item quality: Are the item difficulties reasonably distributed along the latent scale and/or are there items that seem to be redundant, i.e. that do not contribute to calculating the respondent measure?

4. Respondent fit: Are there unexpected respondent response profiles? That is, are there respondents whose answers do not seem to agree with the general structure formed by the large majority of the respondents? This usually means contradictory answers across items.

5. Separation/reliability: Is the item set able to sufficiently separate respondents from each other and thereby reasonably rank the respondents on the latent scale (person reliability) [25].

6. Item reliability: Do the items create a reasonably useful measure [25]?

7. Discrimination: Item discrimination is estimated by the Winsteps program, but not included in the model. This information can be used to investigate whether an item sufficiently discriminates between patients whose measurements are in the neighbourhood of the item's difficulty.

8. Different item functioning (DIF): Are there items that are differently scored by the participants in the three groups? That is, are there systematically different item profiles for the three groups?

## Results

### The Psychological dimension

The items under the heading "*Psychological" *were considered to form a one-dimensional trait. A preliminary Rasch model with all 23 items was set up. An infit/outfit equal to 1.72/3.12 and a negative discrimination for DECID36 gave a strong indication that this item did not belong to the dimension formed by the rest of the items.

A second analysis with 22 items showed a reasonable one-dimensional model, even if a few items showed a slight misfit. Three miss-fitting respondents were found in the second analysis, one from the IA and two from the NCC group. These respondents' answers were very unexpected in relation to their estimate on the psychological scale as indicated by their high infit/outfit. They were put aside in further analysis of this dimension.

A third analysis, now with 22 items and 84 respondents, revealed a not perfect, but reasonable solution. The 3 sub-domains could not be identified. The items' location (measure) on the latent scale is shown in Table 1. The range (-1.86, 1.61) was good, although some items were placed very close to each other on the scale, indicating that such items share the same information from the respective respondent. About 50% of the variation was unexplained. This was also seen in the moderate person reliability (0.83), which means that even if the items were adequate (reliability = 0.95), they were not able to separate the respondents to a sufficient degree. The first contrast disclosed no obvious second dimension, according to the rule of thumb: Unexplained variance in first contrast/Variance explained by items < 0.25 (Table 2). For reasons of comparison, internal consistency was calculated using Chronbach's alpha, showing α = 0.83 (approximate due to non responses).

**Table 1:** Measures of the psychological items, ordered by difficulty

		**MODEL**	**INFIT**		**ESTIM**	
	**MEASURE**	**S.E.**	**MNSQ**	**ZSTD**	**DISCR**	**ITEM**
	1.61	.13	.89	-.8	1.11	MOTH38
	1.33	.11	.90	-.7	1.15	MOTH14
	1.26	.12	.81	-1.4	1.34	FEEL13
	1.20	.12	.90	-.6	1.14	FATH15
	1.14	.13	1.03	.2	.98	FATH39
	.93	.21	.90	-.6	1.10	HAPP25
	.55	.13	.90	-.6	1.10	PROBL37
	.53	.18	.93	-.3	1.03	ANGR26
	.47	.19	1.12	.7	.92	DO44
	.17	.17	1.02	.2	.97	SAD27
	.06	.13	.94	-.3	1.02	THINK42
	-.25	.24	1.15	.7	.91	GET45
	-.31	.13	.98	.0	1.06	BODY20
	-.32	.15	1.39	2.1	.55	TELL43
	-.69	.16	1.20	1.0	.89	HUG22
	-.70	.20	.86	-.7	1.10	SELF19
	-.78	.20	1.08	.5	.91	FRIE16
	-.81	.17	1.00	.0	1.03	FEEL23
	-.86	.17	1.44	1.5	.84	HUG21
	-.88	.17	.97	-.1	1.06	FEEL24
	-1.79	.37	.92	.0	1.03	FATH18
	-1.86	.51	.84	.1	1.12	MOTH17

Mean	0.00	.19	1.01	.0		
Std	.98	.09	.16	.8		
Person reliability = 0.83, Item reliability = 0.95						

**Table 2:** Analysis of residual variances of the psychological dimension

Source	Percent
Variance explained by measures	50.5
Variance explained by persons	19.2
Variance explained by items	31.3
Unexplained variance	49.5
Unexplained variance in first contrast (a possible second dimension)	7.1 (14.4% of 49.5)

The questions were worded quite similarly, which should imply a rating scale approach according to model a. This was tried, but the items behaved very differently. A mixed model with a large set with common category thresholds and a few 'free' items (model b) yielded a somewhat better result in terms of explained variance, but did not fit. We decided to stay with model a, presented in Table 1, with separate scales, and to draw conclusions from there.

**Figure 1:**
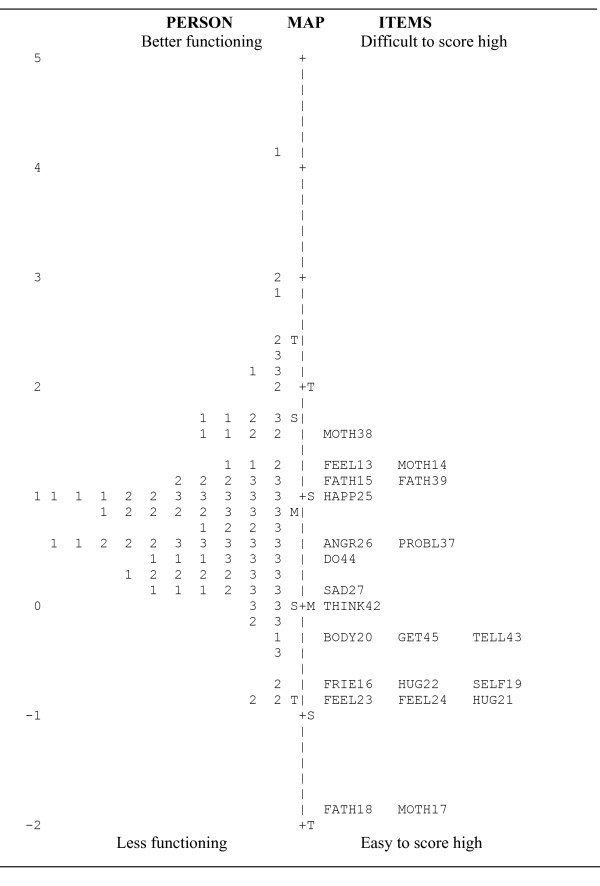
**The Rasch model based on 22 psychological items and 84 children.** M= mean, S= 1 std, T= 2 std. Note: Each number represents one participant, 1= Group IA, 2= Group JCA, 3=Group NCC.

The ability of the questionnaire to capture the respondents' psychological status is illustrated in Figure 1. The questionnaire appeared to be somewhat too easy, and the items FATH18 and MOTH17 did not contribute any noticeable information to the intended measure. There was a slight disordering for 10 out of 22 items. In general, the average person measures should increase as the rating scale values increase. This disordering was concentrated to the lower categories, which usually suggests that the category definitions are too narrow or that fewer response alternatives may yield better information. No firm conclusion can be drawn due to the sparse response rates in the low categories.

**Figure 2:**
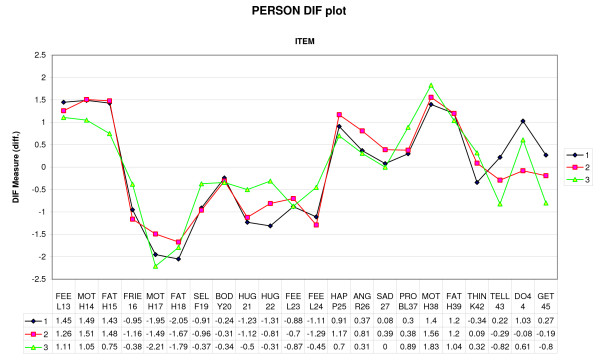
**DIF analyses of the psychological items.** Note: 1= Group IA, 2= Group JCA, 3=Group NCC.

In order to investigate possible differences between the groups, a DIF analysis was performed, shown in Figure 2. No differences were found for the overall psychological measure, p = 0.24 (ANOVA, not shown). However, certain items were scored differently, particularly by the NCC group (Table 3), yielding slightly different profiles for the three groups. The analysis indicated systematically different profiles, p = 0.025, measured by an aggregated Chi^2 ^statistic.

**Table 3:** DIF class specification for the 3 groups on the psychological items

**PERSON**	**SUMMARY DIF**			**ITEM**
Groups	CHI-SQUARE	D.F.	PROB.	Number Name
3	1.3194	2	.5138	1 FEEL13
3	3.7195	2	.1532	2 MOTH14
**3**	**7.9106**	**2**	**.0187**	**3 FATH15**
3	2.8255	2	.2402	4 FRIE16
1	.0000	0	1.0000	5 MOTH17
3	.1233	2	.9421	6 FATH18
3	1.9790	2	.3681	7 SELF19
3	.0987	2	.9537	8 BODY20
3	3.9164	2	.1387	9 HUG21
3	4.4501	2	.1061	10 HUG22
3	.2333	2	.8914	11 FEEL23
3	4.9313	2	.0833	12 FEEL24
3	.8711	2	.6449	13 HAPP25
3	1.6945	2	.4250	14 ANGR26
3	.9765	2	.6113	15 SAD27
0	.0000	0	1.0000	16 DECID36
3	4.1831	2	.1213	17 PROBL37
3	1.8951	2	.3841	18 MOTH38
3	.3453	2	.8422	19 FATH39
3	3.6857	2	.1558	20 THINK42
**3**	**7.0313**	**2**	**.0290**	**21 TELL43**
**3**	**5.8036**	**2**	**.0538**	**22 DO44**
3	3.1978	2	.1991	23 GET45

Three items, scored differently by the groups, are shown in boldface

Total Chi2 = 61 d.f. = 42 p = 0.025

### The Social dimension

Twelve items (see Appendix: Table 7) were considered to constitute a "*Social dimension*", where a high score represented good social functioning. In a preliminary analysis, the parsimonious model (a) seemed to work reasonably, with the exception of item BULLY31 and TOGETH32, which required their own category estimates (model b). The item TOGETH32 showed a high infit as well as a z statistic > 3.3. Together with a low estimated discrimination, this indicated that this item did not contribute very much to the intended estimate of the measure. Therefore, item TOGETH32 was deleted and a new model with 11 items was re-estimated.

Item measures are presented in Table 4. The distribution was narrower than desired, range (-0.56, 0.87) and items FRIEND6 and ACTI10 were very close, indicating that one of them tended to contain redundant information. There was still a considerable amount of unexplained variation, 67%; see Table 5. The variance explained by the 11 items was insufficient, 11%, but there was no clear indication of any secondary dimension, and the first contrast showed 12.4%.

**Table 4:** Measure of the social items, ordered by difficulty

	**MODEL**		**INFIT**		**ESTIM**	
	MEASURE	S.E.	MNSQ	ZSTD	DISCR	ITEM
	.87	.11	1.12	.8	1.00	SHOW8
	.65	.12	.63	-2.4	1.13	DECI29
	.34	.13	.93	-.3	.99	TEAS30
	.19	.14	.98	.0	.87	FRIEN28
	.17	.14	.96	-.2	.90	SCHOO4
	-.11	.15	1.16	.8	1.06	GYMN7
	-.27	.16	1.23	1.0	1.11	BREAK9
	-.36	.20	1.27	.9	.97	BULLY31
	-.45	.18	.70	-1.4	1.18	FRIEND6
	-.46	.18	1.23	1.0	.95	ACTI10
	-.56	.18	1.46	1.8	.83	TEACH5

Mean	0.00		1.06	0.2		
Std	0.46		0.24	1.2		
Person reliability = 0.52, Item reliability = 0.87						

**Table 5:** Analysis of residual variances of the social dimension

Source	Percent
Variance explained by measures	33.1
Variance explained by persons	22.1
Variance explained by items	11.0
Unexplained variance	66.9
Unexplained variance in first contrast (a possible second dimension)	12.4 (18.5% of 66.9)

**Figure 3:**
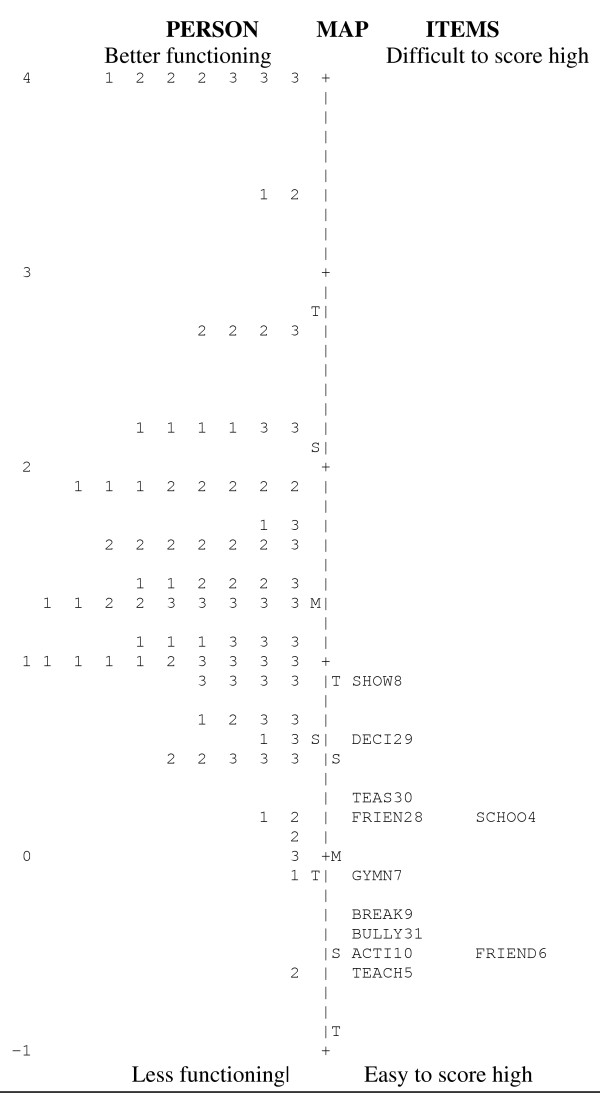
**The Rasch Model based on 11 social items and 87 children.** M= Mean, S= 1 std, T= 2 std. Note: Each number represents one participant, 1= Group IA, 2= Group JCA, 3=Group NCC.

An item reliability = 0.87 indicated that the 11 items constituted a reasonable dimension, as intended, but the person reliability = 0.52 implied that the item set was not able to sufficiently separate the respondents on the latent (social) scale. The ability of the questionnaire to capture the respondents' social status is illustrated in Figure 3. It can be concluded that the item set did not meet the target study sample, which is a partial explanation for the weak person reliability. From a 'difficulty' point of view, the questions were 'too easy'. For reasons of comparison, Chronbach's alpha was used to test the internal consistency and showed: α = 0.80 (approximate due to non responses).

**Figure 4:**
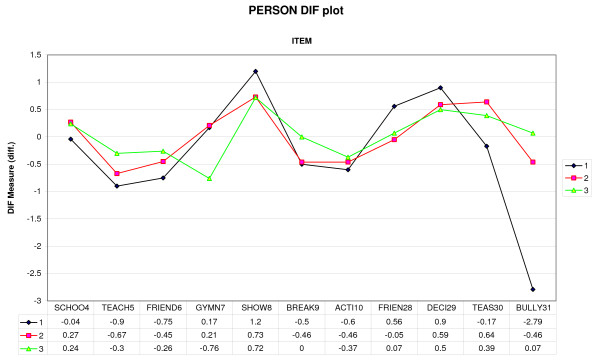
**DIF analysis of the social items.** Note: 1= Group IA, 2= Group JCA, 3=Group NCC.

Figure 4 and Table 6 indicate that the three groups scored the items roughly similarly, except for Gymn7, group 3 ≠ group 1, 2 p = 0.038), and Bully31 (group IA had only extreme values). The items, as they were formulated, were not able to build a sufficient social dimension, and were thus not able to reveal potential, overall group differences, if any.

**Table 6:** DIF class specification for the 3 groups on the social items

PERSON	SUMMARY DIF			ITEM
Groups	CHI-SQUARE	D.F.	PROB**.**	Number Name
3	.8312	2	.6581	1 SCHOO4
3	1.7952	2	.4039	2 TEACH5
3	1.1539	2	.5588	3 FRIEND6
**3**	**6.5071**	**2**	**.0378**	**4 GYMN7**
3	3.9308	2	.1377	5 SHOW8
3	2.1503	2	.3376	6 BREAK9
3	.2749	2	.8728	7 ACTI10
3	3.4232	2	.1778	8 FRIEN28
3	1.9843	2	.3671	9 DECI29
3	4.5712	2	.0998	10 TEAS30
2*	3.1456	1	.0761	11 BULLY31
0	.0000	0	1.0000	12 TOGETHER

One item, differently scored by the groups, is in boldface

* The IA children are not included, due to zero variance.

All children answered '5'

Total Chi^2 ^= 29.8 d.f. = 21, p = 0.096

**Table 7:** Appendix: Items/Questions in IAPSQ

**No:**	**Item name**	***Psychological ***
		*Emotional*

1	FRIE16	How much do your friends love/like you?

2	MOTH17	How much does your mother love you?

3	FATH18	How much does your father love you?

4	SELF19	How much do you love/like yourself?

5	BODY20	How much do you like your body?

6	HUG21	How do you like being hugged by your mother?

7	HUG22	How do you like being hugged by your father?

8	FEEL23	How do you feel in general?

9	FEEL24	How will you feel when you become grown-up?

10	HAPP25	How often do you feel happy?*

11	ANGR26	How often do you feel angry?

12	SAD27	How often do you feel sad?

		*Emotional/Cognition*

13	FEEL13	How do you feel when you think about your condition?

14	MOTH14	How do you think your mother feels when she thinks about your condition?

15	FATH15	How do you think your father feels when he thinks about your condition?

16	PROBL37	Do you think about your condition?

17	MOTH38	Does your mother think about your condition?

18	FATH39	Does your father think about your condition?

19	THINK42	Do you think about your body?

		*Self-determination*

20	DECID36	How much can you decide about your condition at home?*

21	TELL43	Do you say what you really want?*

22	DO44	Can you do as you like?*

23	GET45	Do you get what you want?*

		***Social ***

1	SCHOO4	How do you like school?

2	EACH5	How is your relationship with the teacher?

3	FRIEND6	How is your relationship with friends?

4	GYMN7	How do you like physical activity at school?

5	SHOW8	How do you like taking a shower after physical activity?

6	BREAK9	How do you like the breaks at school?

7	ACTI10	How do you like activities after school?

8	FRIEN28	How often are you together with friends?*

9	DECI29	How much can you decide when you are with friends?*

10	TEAS30	Have you been teased at school?

11	BULLY31	Have you been bullied at school?

12	TOGETH32	Do you have a best friend and if you have a best friend, how often are you together*?

* Reversed items

### Face and Content validity

The prior exploratory interviews with patients/adolescents with imperforate anus and their parents were the basis for creating the IAPSQ, and this procedure involved face and content validity. Content validity was established by having specialists in paediatric surgery and in child and adolescent psychiatry construct the questions for both the interviews and the IAPSQ.

### Feasibility

Altogether, almost half (43/87) of all children participating in the present study commented on what they thought about the questions posed in the IAPSQ. The comments were both positive, i.e. *fun to answer*, and negative, i.e. *difficult and odd questions*. In the IA group, 72% of the children commented on the questions. In Comparison Group I (JCA), 50% of the children commented on the questions, and in Comparison Group II (NCC), the corresponding figure was 31%.

## Discussion

The present study describes the construction and psychometric properties of the IAPSQ, a self-report questionnaire designed to evaluate psychosocial functioning in children with IA. Previous examination of the questionnaire's reliability and validity used CTT, i.e. Chronbach's alpha. Because more modern techniques, such as the IRT approach, are thought to be more informative than assessments obtained from the CTT [26] and as factor analyses treat data as interval data, a supplementary and more comprehensive validation of the IAPSQ has been achieved using the IRT/Rasch model.

Overall, the findings of the Rasch analysis revealed the sound psychometric properties of the IAPSQ, although some deficiencies were identified. The psychological dimension can be regarded as reasonable. The social dimension showed almost satisfactory item reliability. However, the person reliability of the social dimension did not discriminate adequately and should be expanded for future purposes so as to be more in balance with the target population.

The two latent domains were constructed as intended, see "Questionnaire construction procedure", and comprised 35 items. The total set of these items could as such have been analysed for intended latent dimensionality. As there were only 87 respondents, divided into three groups, this was not done due to the risk of obtaining artificial dimensions, which would be difficult to verify.

### The Psychological dimension

The psychological dimension consisted of several items, and it would seem to include items appropriate to demonstrating a relevant emotional concept. Except for one item, the psychological items fit the model and seem to support unidimensionality. Unidimensionality is an essential marker of construct validity [27], that is, the extent to which self-report scores indicate the theoretical construct of interest [28,29].

Initially, the psychological dimension was expected to consist of different aspects, and it was therefore divided into three sub-dimensions featuring different emotional issues. However, the children's answers did not disclose such a differentiation, which was confirmed by the analysis. This was the rationale for the simplification, thus far of just one dimension.

In such a small study, the analysis could be influenced by some misfit respondents, and it was revealed that 3 participants scored unexpectedly on the psychological items. It is recommended that such individuals be set aside in the estimation process, thus avoiding misleading models. We will never know the reason for these "unexpected" scores, thus whether the children rated these items in an odd manner on purpose or by chance. The respondents may not have understood the question, they may not have been concentrated on the task, or they may have had special experiences, etc. However, the items that were unexpectedly scored were related, and our assumption is that the children had their reasons for responding as they did. When a questionnaire is used in a practical application, such participants must be taken into consideration, regardless of how they have scored [30].

In the psychological dimension, one item was found to be a misfit: DECID36, *How much can you decide about your condition at home? *This item was supposed to fit in with the other psychological items, but the analysis strongly suggested its removal. DECID36 may belong to another domain, and one solution may be to try to tie it in with items connected to "experiences of care", which are not pertinent to the present study.

One notable finding was how items FATH18 and MOTH17 tended to score high. These items, *how much does your mother *and *how much does your father love you*? may seem natural and hopeful, but they did not contribute to the intended psychological scale. If FATH18 and MOTH17 are to be kept in the IASPQ, they should be reformulated or replaced with other relevant items. There were small differences between the profiles of the three groups on the psychological dimension. This may be a consequence of the limited study size. It is possible that further questions should be added that would make the questionnaire more sensitive to potential group differences.

### The social dimension

After removal of the misfitting item TOGETH32, the social domain ended up with 11 items. Item reliability was found to be sufficient. However, these 11 social items did not capture the intended dimension; person reliability was unsatisfactory. One reason for the low person reliability may simply be the small number of items in the social dimension. Another reason may be that the social items were too easy to score high. Our general intention was to use a hopeful approach in relation to the children and to pose the questions in a positive manner. We wanted the questionnaire to appear attractive and to encourage interested children to answer the questions. Our ambition was also to create gentle ethical questions and not to embarrass the children with IA, whose condition is associated with shame.

However, there may be evidence that the social items are relevant and that we are on the right track, as item reliability was considered sufficient. A study from 2003, using IRT on a well-established self-report questionnaire on psychopathology, showed that only a few social items discriminated well across different scale levels. The valid social items from that study are similar to some of the social items in the IAPSQ [31].

Social item (no. 11) asking whether the *child had been bullied *is worthy of attention. This item received extremely high scores from the children with IA. All children with IA answered on the highest possible level, indicating that they had never been bullied. Identical answers from a group of respondents usually indicates the inadequacy of the question posed or the anchor words used [25].

Thus, item difficulty in the social dimension must be strengthened, as must a few other items in the IAPSQ. Some of the questions should be reformulated, and an increased number of items in the social dimension is needed as well. Nevertheless, it is expected that questionnaire items will be adjusted following assessments of validity using IRT. For instance, when the psychometric properties of the SDQ were evaluated, it was recommended that some items be revised and reanalysed [32].

### Face and content validity

Both face and content validity appear to be verified in the IAPSQ. Content validity is considered crucial when developing an instrument [33]. The first and most important step to ensure content validity during the construction of the IAPSQ was the initial explorative interview study. The interviewed patients/adolescents, who had severe consequences of imperforate anus, and their parents were also encouraged to add any other questions of concern. Interviews have been used in the process of constructing quality of life questionnaires to ensure content validity [34-36].

Another important aspect of ensuring face and content validity was the panel of professional experts who created the questions for the initial interviews and in the next step for the questionnaire. An additional stage of establishing validity of the IAPSQ was the pre-testing of the questionnaires. Items whose meaning was not perfectly clear were identified, and then modified and clarified.

### Feasibility

The IAPSQ seemed to be well accepted by the children in our study, even though they had to answer numerous questions. Unfortunately, we do not know the time required to complete the IAPSQ. Nevertheless, it did not seem to be a problem for the participating children. None of the study participants commented on the time required. The children appeared to have accepted the questionnaire; the reliability of reports made by children 8–11 years would seem to be quite good [37].

Many of the respondents provided a final comment on the questions in the questionnaire, and more IA children commented than did children in the comparison groups. This finding may signify the engagement of the IA children, that the survey primarily addressed them. We know nothing about how independently the children completed the questionnaires. Our impression is that most children filled in the IAPSQ by themselves, also considering the children's comments (see above). However, one IA mother confirmed that she had helped her child complete the questionnaire.

### Strengths and limitations

A strength of the present study is the inclusion of two comparison groups; a healthy group and a group with a chronic condition. This measure was taken to create a questionnaire of sufficient range so possible group differences (in terms of the two dimensions), can be established. The use of a transformed sum score (the Rasch approach) is also a strength. The Rasch approach allows arithmetic operations and valid evaluation of item and group characteristics that had not been possible by using the raw sum score approach.

In such a small study, complicated models should be avoided due to the risk of over-fitting and misleading conclusions. A simple Rasch model, with relatively few parameters and a straightforward estimation of the difficulty parameter, was chosen. It is also commonly recommended to use the Rasch modelling approach in small studies. The method is not thought to develop a certain model, but rather to reveal its rough character from what is observed. Results from a Rasch analysis are considered approximate guidelines for taking actions towards improvement of the questionnaire.

The construction process of the questionnaire might seem time consuming, but it was necessary to achieving our intention and goal: to create a sound self-report questionnaire for children with imperforate anus. On the other hand it may be a strong point that we used and generated content from the patients to formulate the items for the questionnaire. By interviewing children/patients with IA and their parents, invaluable information was received about the experiences of living with the malformation and thus the issues of concern for these patients. This information was operationalized into the questionnaire items by the clinicians. The purpose of constructing the questionnaire was for clinical use in the future, addressing the psychosocial functioning of the children/patients.

The core restriction with this study is the sample size. However the group is representative of the patients with high and intermediate imperforate anus, treated according to the current routines. They constituted almost all available patients, born between 1987 and 1992, treated in the greater Stockholm area and affiliated regions. Twenty- five out of 30 patients (one had moved abroad) represents about one quarter of all patients in the country. Small studies are inevitably carried out and have to be analyzed as they are in order to take reasonable actions at an early stage.

A difficult problem in this small study has been to evaluate the dimensionality. This has to be further investigated with a more elaborated choice of scale. Even if the item reliability is quite good, there is too much unexplained, residual variation. This affects the decision of dimensionality as well as the validity. The Rasch analysis assumes unidimensionality. The analyses did not reveal any obvious further dimensions. The information on validity will be further evaluated when the questionnaire has been improved. In this setting, the choice was to include 'normal children' as well as a group of children with another chronic condition. However, the Rasch approach, as applied in this study, provided some guidance on validity, which has been taken into account when choosing further improvements.

## Conclusion

The present data suggest that the version of the IAPSQ used here provides a reasonably valid and reliable measure of psychosocial functioning for clinical use among children with IA, although some revisions are suggested for the next version. By using the Rasch model, we discovered that specific items (DECID36 and TOGETH32) should be discarded and that other items should be reformulated to make the questionnaire more "on target". The social dimension has to be expanded with further items if it is to reasonably capture a social domain. As regards the psychological dimension, construct validity seems to have been achieved.

A revised IAPSQ needs to be explored on a similar population. Due to the limited availability of patients/respondents with IA, such a study would probably require a multi-centre approach. If possible, a group of children with IA of a similar age would be suitable for testing the revised IAPSQ. It may also be interesting to contact the children in the present study, who are now adolescents/young adults.

## Competing interests

The authors declare that they have no competing interests.

## Authors' contributions

MN designed the study, participated in the statistical analysis, and was the main writer. UB performed the statistical analysis and was involved in writing the manuscript. P-AR was also involved in writing the manuscript, and reviewed the manuscript together with KC.
